# The Use of Testicular Sperm Extraction in Male Infertility Patients Without Azoospermia

**DOI:** 10.7759/cureus.97366

**Published:** 2025-11-20

**Authors:** Moshe Wald

**Affiliations:** 1 Urology, University of Iowa Hospitals and Clinics, Iowa City, USA

**Keywords:** cryptozoospermia, high sperm dfi, ivf, male infertility, testicular sperm extraction

## Abstract

Testicular sperm extraction (TESE) in its various versions has been traditionally used in male infertility patients with azoospermia, with the intention of utilizing surgically retrieved sperm for in vitro fertilization (IVF) with or without intra-cytoplasmic sperm injection (ICSI). Possible risks of TESE include bleeding, infection, and inability to find sperm. Microdissection TESE, a more extensive type of surgical sperm retrieval procedure, may be associated with a transient post-operative decline in serum testosterone levels. More recently, the possibility of using TESE in non-azoospermic infertile men has been gaining attention. However, heterogeneity still exists in the available data. Older studies reported limited clinical benefits for the utilization of testicular over ejaculated spermatozoa, while newer publications suggest more promising outcomes for the utilization of surgically retrieved sperm for ICSI in non-azoospermic patients. Suggested indications for TESE in non-azoospermic infertile men included failure of IVF cycles utilizing ejaculated sperm, high sperm DNA fragmentation index, cryptozoospermia, and necrospermia. This editorial provides insight into the rationale, potential candidates and benefits for the consideration of TESE in non-azoospermic infertile men.

## Editorial

Overview of procedure

**Figure 1:**
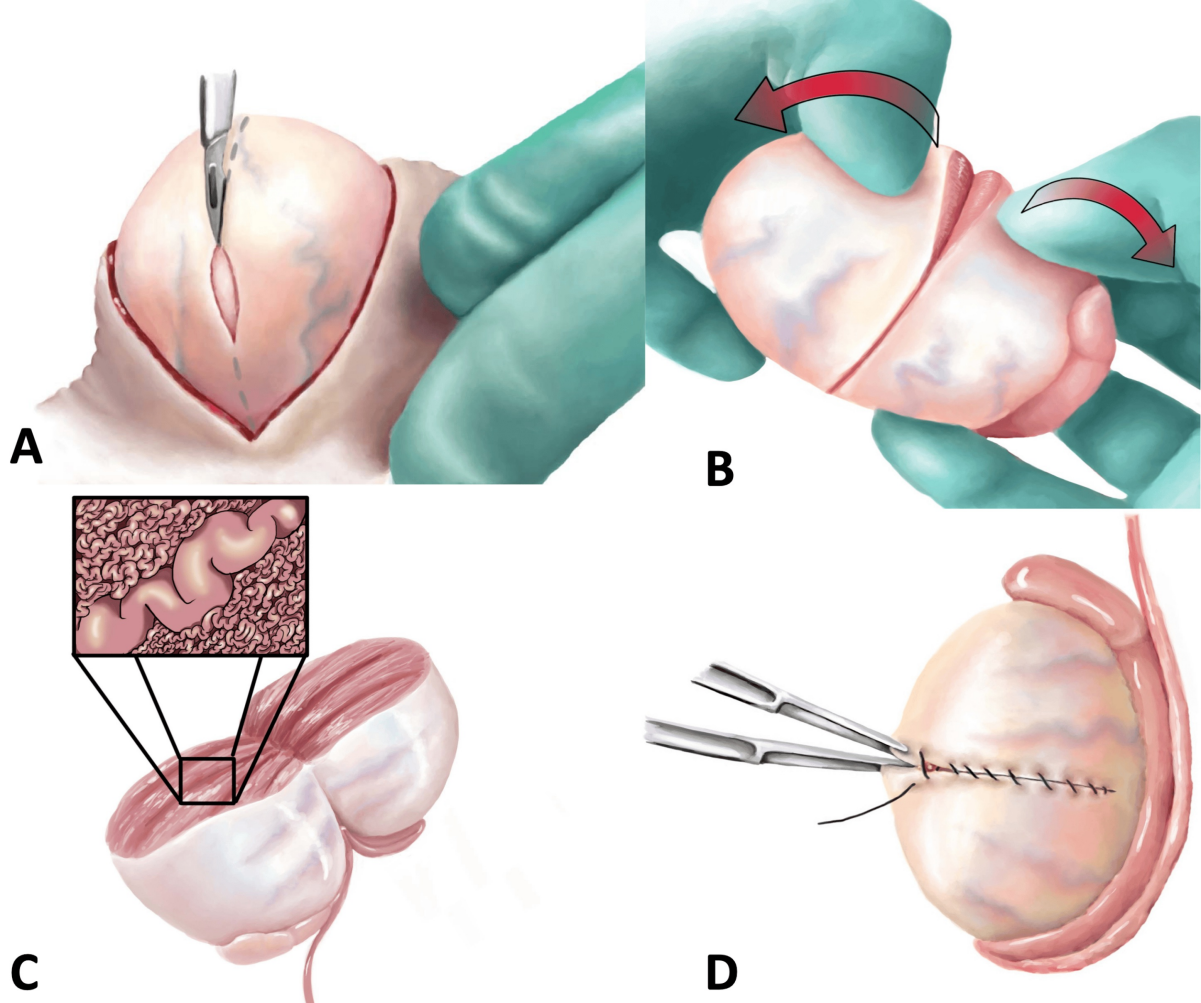
Microdissection Testicular Sperm Extraction (MicroTESE) A: The testicle is delivered through scrotal incision and an equatorial incision is made in the tunica albuginea. B: The testicle is bivalved, exposing seminiferous tubules. C: Seminiferous tubules are carefully searched under an operating microscope until a dilated tubule is identified. These dilated tubules are more likely to contain sperm and should be harvested to be processed by the embryology team and examined under a microscope. D: Once microTESE is complete, hemostasis is achieved with bipolar cautery and tunica albuginea is closed with a running non-absorbable monofilament suture. The testicle is placed back in the scrotum, and tunica vaginalis, dartos and skin layers are closed. Source: [2]. Reprinted with permission from American Urological Association Education and Research, Inc.

Testicular sperm extraction (TESE) is a surgical sperm retrieval procedure that is typically offered to men with infertility and azoospermia. Surgically retrieved sperm could then be used either fresh or after being cryopreserved for in vitro fertilization. The latter may involve intra-cytoplasmic sperm injection (ICSI), at the discretion of the treating Reproductive Endocrinology team. TESE is typically performed as an outpatient procedure in the operating room, under either deep sedation or general anesthesia, depending on the type of azoospermia (obstructive or non-obstructive) and the anticipated extent of the procedure. In cases of obstructive azoospermia (for example, following a past vasectomy), TESE may be limited to a small incision in the tunica albuginea of one testicle, allowing for harvesting a small portion of testicular tissue, from which sperm can be extracted. However, in cases of non-obstructive azoospermia (NOA) (such as Klinefelter Syndrome), microdissection TESE, a more extensive version of this surgery, is often used (Figure 1). Microdissection TESE typically involves bivalving one or both testicles, allowing for an extensive search performed under an operating microscope, in an attempt to identify individual seminiferous tubules that exhibit characteristics suggestive of spermatogenesis, such as a wider diameter [1]. 

While rates of successful surgical sperm retrieval for TESE in cases of obstructive azoospermia are obviously very high, NOA cases, in which spermatogenesis is impaired, pose a challenge for the surgical procurement of sperm. With the implementation of microdissection TESE, surgical sperm retrieval rates in NOA were reported to increase from 16.7-45% to 42.9-63% [1]. In addition to the risk of inability to find sperm, microdissection TESE also carries a low risk for bleeding and infection. Serum testosterone might decline after microdissection TESE but has been reported to improve to 80.6% - 93.1% of the preoperative level at 12 months from surgery. 

Why use TESE in non-azoospermic patients?

In general, the rationale for using sperm that were surgically retrieved from the testicles (as opposed to ejaculated spermatozoa) is based on the concern for oxidative stress and nuclear DNA damage that spermatozoa might sustain during passage through the male genital tract, which in turn could potentially impair ejaculated sperm quality and lead to poor IVF/ICSI outcomes. Another potential concern has been raised in cases of cryptozoospermia, in which repeated centrifugations are required to obtain sperm. Such repeated centrifugations might increase reactive oxygen species production and eliminate antioxidants from the seminal fluid, with subsequent negative effects on sperm quality and IVF/ICSI outcomes [3].

Non-azoospermia conditions in which TESE could be considered

Several studies have investigated the utilization of TESE in certain clinical scenarios in which the male partner is not azoospermic.

Failed ICSI cycle with ejaculated sperm

Benchaib et al. assessed the outcomes of ICSI using sperm obtained via TESE in couples who had a failed ICSI cycle with ejaculated sperm, compared to the outcomes of a second ICSI cycle using ejaculated sperm. The live birth rate was higher in the TESE group (22.2% vs. 0.0%, p < 0.001). Univariate analysis on parameters of the failed first ICSI cycle identified teratozoospermia, cryptozoospermia and a high DNA fragmentation index (DFI) as prognostic factors to consider surgically retrieved testicular sperm for repeat ICSI cycle after failure of first ICSI with ejaculated sperm [4].

Cryptozoospermia

A meta-analysis published in 2016 compared ICSI outcomes with either surgically-retrieved testicular or ejaculated sperm in men with cryptozoospermia and included five cohort studies that encompassed 272 ICSI cycles. While there was a significant trend toward increasing maternal and paternal age in the group that used testicular sperm, there were no differences in ICSI pregnancy or fertilization rates between testicular and ejaculated sperm groups. That study concluded that the reviewed literature did not support a recommendation for men with cryptozoospermia to prefer the usage of testicular sperm over ejaculated sperm for ICSI [5].

High sperm DNA fragmentation index

A past review by Awaga et al. assessed the potential merit of TESE in certain non-azoospermic conditions. These authors reported that in a prospective study on men with high sperm DFI and oligospermia, the probability of live births after IVF/ICSI was significantly higher with testicular compared to ejaculated spermatozoa, but that such a benefit for testicular sperm was not found in a retrospective study in men with high DFI only. Additionally, testicular and ejaculated sperm used for IVF/ICSI yielded similar clinical pregnancy rates in a randomized controlled trial in men with asthenospermia with or without teratospermia, as well as in a retrospective study in men with isolated asthenospermia [3].

Necrospermia

Surgery or trauma involving the male reproductive organs might lead to the formation of anti-sperm antibodies, which could potentially be associated with necrospermia. The absence of motile sperm in the ejaculate would compromise the utilization of ejaculated sperm for IVF/ICSI. TESE may at least partially overcome the negative effect of the anti-sperm antibodies by retrieving sperm before they reach the seminal fluid, where they would be exposed to the antibodies, as suggested by Chavez-Badiola et al. In that study, TESE reportedly yielded motile sperm in a patient who underwent vasectomy reversal and was found to have anti-sperm antibodies and necrospermia. The motile surgically retrieved sperm subsequently fertilized oocytes in an ICSI cycle [6].

Summary

Data have been accumulating regarding the possible use of TESE in infertile men who do not have azoospermia, suggesting potential benefit for the utilization of surgically retrieved sperm in certain non-azoospermic conditions. Older reports did not find an advantage for the usage of testicular sperm rather than ejaculated sperm for ICSI in men with cryptozoospermia, and suggested that the clinical benefits of using testicular rather than ejaculated spermatozoa (in terms of higher probability of pregnancy) are limited to men with high DFI and oligospermia. 

However, newer studies published since that time provide additional data, which suggest more promising outcomes for the utilization of surgically retrieved sperm for ICSI in non-azoospermic patients, and also identified teratozoospermia, cryptozoospermia and a high DFI as prognostic factors to consider surgically retrieved testicular sperm for repeat ICSI cycle after failure of first ICSI with ejaculated sperm.

Additional studies may shed more light on the role of TESE in infertile men with necrospermia as well as in other non-azoospermic conditions involving impairment in sperm quantity or quality.
